# Innovationen im Ostseeraum: Nils Alwall, Lembit Norviit, Adolfs Martins Steins und die künstliche Niere

**DOI:** 10.1007/s00120-023-02239-6

**Published:** 2023-12-13

**Authors:** Nils Hansson, Juris Salaks, Friedrich H. Moll, Thorsten Halling, Erki Tammiksaar

**Affiliations:** 1 Institut für Geschichte, Theorie und Ethik der Medizin, Centre for Health and Society, Medizinische Fakultät, Heinrich-Heine-Universität Düsseldorf, Moorenstr 5, 40225 Düsseldorf, Deutschland; 2 Institut für Geschichte der Medizin, Rīga Stradiņš Universität, Riga, Lettland; 3 Centre for Science Studies, Estonian University of Life Sciences, Tartu, Estland

**Keywords:** Medizin im Ostseeraum, Dialyse, Wissenstransfer, Kalter Krieg, Urologie, Medicine in the Baltic Sea region, Dialysis, Transfers of knowledge, Cold War, Urology

## Abstract

Die Konstruktion einer der ersten in der Praxis erfolgreich eingesetzten Dialyseapparate ist in der Medizingeschichte mit dem Wirken des schwedischen Mediziners Nils Alwall fest verbunden. Zusammen mit Kollegen entwickelte er in den 1940er-Jahren einen Dialysator, der die Kombination von Dialyse und Ultrafiltration mit Membranen (Cellophan-Schläuchen) umsetzen konnte. In die fachkulturelle Erinnerung sind die beteiligten Lembit Norviit aus Estland und Adolfs Martins Steins aus Lettland, beide Mitautoren des einflussreichen *The Lancet*-Artikels „Clinical extracorporal dialysis of blood with artificial kidney“ (1948) bisher nicht eingegangen. Am Beispiel dieser Zusammenarbeit kann der Wissenstransfer zwischen estnischen, lettischen und schwedischen Forschern dargestellt werden.

## Abstract (en)

In connection with the construction of one of the first practical dialysis machines, medical historians emphasize the work of the Swedish physician Nils Alwall. Together with his colleagues, he developed a device in the 1940s that could implement the combination of dialysis and ultrafiltration with membranes (cellophane tubes). Little known is the involvement of the physicians Lembit Norviit from Estonia and Adolfs Martins Steins from Latvia, both coauthors of the influential research article *Clinical extracorporeal dialysis of blood with artificial kidney *that was published in *The Lancet* in 1948 and the transfer of knowledge between Estonian, Latvian and Swedish researchers.

## Einleitung

Rückblickend beschrieb der schwedische Nephrologe Nils Alwall (1904–1986) im Jahr 1985, im Rahmen eines Nephrologenkongresses in Rottach-Egern, die Therapiemöglichkeiten bei fortgeschrittener Niereninsuffizienz in der Zeit bis zum Zweiten Weltkrieg:

*„When I started working at our University Medical Department in the mid-1930s, the treatment of uremia followed the traditional European or even international pattern: bed rest and diet. The uremic patient suffered from sickness, poor appetite and vomiting. The therapy was largely palliative to relieve the patients’ suffering. The possibility of dialysis was not mentioned in the textbooks“* [1].

In der Einleitung ihrer *The Lancet*-Publikation „Clinical extracorporeal dialysis of blood with artificial kidney“ aus dem Jahre 1948 schildern Alwall und 2 Koautoren, dass bereits mehrere andere Forscher versucht hätten, ein für die klinische Praxis taugliches Gerät für die Blutreinigung außerhalb des Körpers zu konstruieren [2]. Dabei erwähnen sie an erster Stelle einen Apparat des Niederländers Willem Kolff (1911–2009), der laut den Autoren allerdings unerwünschte und z. T. tödliche Nebenwirkungen gehabt habe, wie Hämolyse, Schock und Lungenödem. Das Forscherteam aus Lund dagegen habe das von ihnen entwickelte Gerät seit mittlerweile 3 Jahren erfolgreich klinisch eingesetzt.

Im Zusammenhang mit der Entwicklung einer der ersten praktikablen Dialyseeinheiten gilt das Wirken des schwedischen Mediziners Nils Alwall als zentral [3]. Alwalls „künstliche Niere“ wurde zum Grundstein des 1964 durch Holger Crafoord (1908–1982) gegründeten Medizintechnikunternehmens Gambro, später weltweit führend für Produkte der Dialysebehandlung. Nahezu unbekannt ist die Beteiligung an den frühen Entwicklungen in den späten 1940er-Jahren der beiden anderen Autoren des *The Lancet*-Beitrags, der Ärzte Lembit Norviit (1913–1967) aus Estland und Adolfs Martins Steins (1911–1996) aus Lettland [4]. Aktuelle Darstellungen zur Geschichte der künstlichen Niere fokussieren auf Nils Alwall und inszenieren die Entwicklung der ersten Dialyseapparate als einsames Experimentieren im Keller einer alten internistischen Klinik in Lund [5]. Anhand von bisher wenig beachteten Archivalien aus Tallinn, Tartu, Riga und Lund untersuchen wir in diesem Beitrag die Gründe für die weitgehende Unsichtbarkeit von Norviit und Steins sowohl in der Medizingeschichte als auch im fachkulturellen Gedächtnis von Nephrologie und Urologie. Diskutiert wird am Beispiel der Entwicklung der Dialyse auch die Frage des Wissenstransfers im Ostseeraum während des Kalten Krieges [6].

## Alwall und die deutsch-deutsche Urologie und Nephrologie

**Abb. 1:**
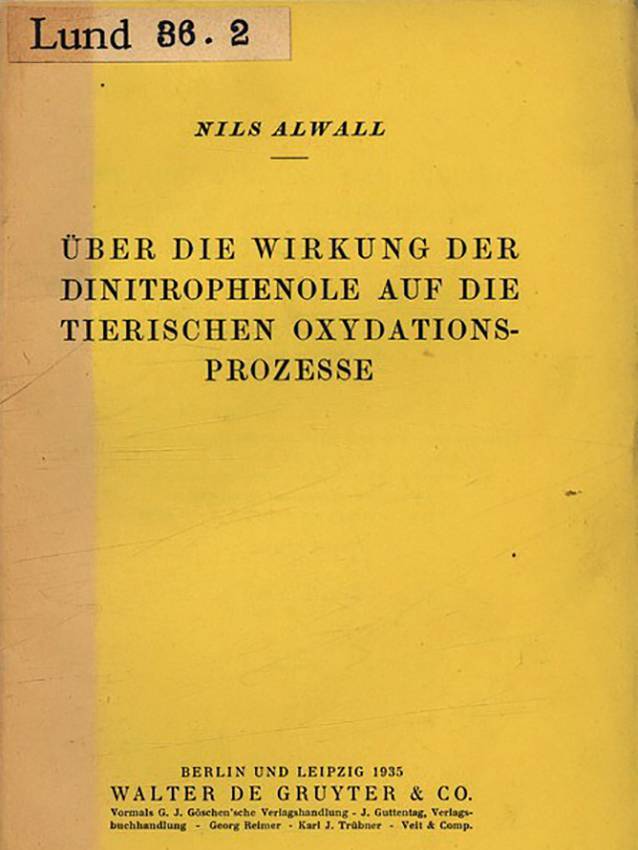
Promotion Nils Alwalls (1904–1986) in deutscher Sprache, bei dem auch in der Medizin renommierten Walter de Gruyter-Verlag, Berlin-Leipzig, 1935 erschienen. (Repro Moll, mit freundl. Genehmigung)

Nils Alwall, 1904 als Sohn einer nicht wohlhabenden Bauernfamilie im südschwedischen Kristianstad geboren, begann mit 17 Jahren sein Medizinstudium in Lund. 1926 wurde er unbezahlter Mitarbeiter am Physiologischen Institut, bevor er sich 1929 der Pharmakologie zuwandte. Nach dem Ersten Weltkrieg war die Tätigkeit in Grundlagenwissenschaften wie Physiologie oder Pharmakologie eine wichtige Grundlage für eine akademische Karriere in der Medizin. 1936 legte er seine Promotion ab [7], und wie bis 1941 in Schweden üblich, verfasste er seine Dissertationsschrift in deutscher Sprache ([8]; Abb. 1).

Während sich die jüngere Generation schwedischer Mediziner nach Ende des Zweiten Weltkriegs verstärkt der angloamerikanischen Wissenschaftscommunity zuwandten, waren es einerseits die weit verbreiteten Deutschkenntnisse und andererseits die politische Neutralität des Landes, die die bis in die späten 1930er-Jahre mit und in der deutschen Wissenschaft sozialisierten schwedischen Mediziner für beide deutschen Staaten besonders attraktiv machten [9].

**Abb. 2:**
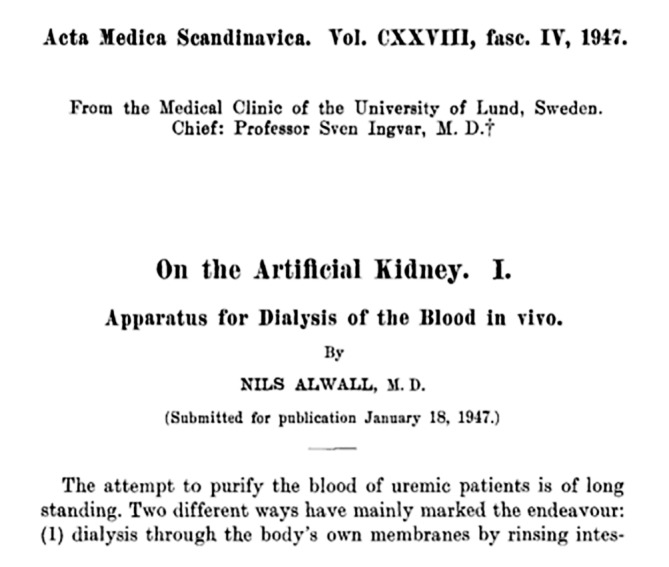
Ausriss aus *Acta Med Scan* Vol 128 Bd IV 1947 317–325, in dem zum ersten Male die Alwall-Niere beschrieben wurde. (Repro Moll-Keyn, mit freundl. Genehmigung)

Nach einem Forschungsaufenthalt in Ungarn 1935–1936 an der Universität in Pécs wandte er sich der Inneren Medizin in Lund zu. Gleichzeitig setzte er seine Forschungsarbeiten zur Ultrafiltration fort [10]. Früh hatte er auch begonnen, in Zusammenarbeit mit der Industrie eine Dialysetrommel zu entwickeln (Avesta Steel Company; [11]). Am 4. September 1946 führte Alwall bei einem urämischen Patienten mit einer Silikose die erste Hämodialyse aus, doch erbrachten die ersten Anwendungen am Menschen nur einen kurzen Überlebensvorteil. Erst bei Patienten mit einem akuten Nierenversagen hatte die neue Methode durchschlagenden Erfolg [12]. 1947 publizierte er erstmals seine Überlegungen zu einem extrakorporalen Dialysegerät (Abb. 2; [13]).

In den Jahren nach den weit rezipierten Publikationen von 1947 und 1948 reiste Alwall um die Welt, um die theoretischen Grundlagen und die praktischen Ergebnisse seiner Forschungen zu präsentieren. Auf Einladung des damaligen Präsidenten der Deutschen Gesellschaft für Urologie, Ferdinand May (1898–1978), führte ihn seine erste Auslandsreise 1949 zum Urologenkongress nach München, wo auch der bekannte Frankfurter Internist und Nephrologe Franz Volhard [14] im Publikum saß. Dem Alwall-Biographen Håkan Westling zufolge war Volhards Urteil nach dem Vortrag knapp, aber eindeutig: „Die schwedische Methode ist die richtige“ [15].

Ganz so eindeutig fiel das Urteil nicht bei allen Kollegen aus. Sie kritisierten Westling zufolge einerseits, dass die Behandlung theoretisch nicht genug untermauert sei, andererseits, dass die praktischen Erfahrungen noch zu spärlich wären. Es sollte noch dauern, bis das Konzept sich großflächig etablierte. Entscheidend dafür war die Begegnung Alwalls mit dem schwedischen Unternehmer Holger Crafoord 1961, der, wie bereits erwähnt, 3 Jahre später das Unternehmen Gambro gründete. Vermutlich trug die internationale Sichtbarkeit Alwalls im westlichen und östlichen Europa dazu bei, dass die Lunder Medizinische Fakultät Alwall im Jahre 1957 zum „Professor of Medicine, especially Nephrology (personal chair)“ ernannte [16]. Seine Abteilung wurde – so betont sein ehemaliger Schüler Carl M. Kjellstrand in seinem Nachruf auf Nils Alwall aus dem Jahr 1985 – in den folgenden Jahrzehnten zu einem internationalen führenden Zentrum der klinischen Nephrologie für Forscher aus Ost- und Westeuropa, Amerika und Nordafrika [17].

Im Universitätsarchiv Lund sind neben Briefen zwischen Alwall und Volhard auch ein 4‑seitiges Manuskript („Franz Volhard und die aktive Nierentherapie während seiner letzten Lebensjahre“) aufbewahrt, das Alwall auf einer Tagung aus Anlass des 100. Geburtstags von Volhard 1972 in Halle vorgetragen haben soll. Es zeigt, dass die Begegnung mit Volhard auch mehr als 20 Jahre später Alwall im Gedächtnis geblieben war:

*„Es wäre vermessen von mir hier auf deutschem Boden ein Bild von Volhards Leistungen in der Medizin und besonders in der Nephrologie zeichnen zu wollen. Als wenn ein Sperling am Tanze der Kraniche teilnehmen würde, wie es in einem schwedischen Sprichwort heisst. Mein Beitrag zur Gedächtnisfeier besteht aus einigen Briefen von Volhard vom Ende der vierziger Jahre und persönlichen Eindrücken von einem der medizinischen Grössen dieser Zeit beim Urologenkongress in München im September 1949. Volhard war damals 77 Jahre alt. Es war meine erste Auslandsreise nach dem Kriege. Die Dialyse wurde zu diesem Zeitpunkt allgemein, jedenfalls unter den Internisten, als ineffektiv und sinnlos angesehen, an der äussersten Grenze des ethisch und juristisch Zugelassenen“* [18].

Nach detaillierten Referaten der Volhard-Alwall-Korrespondenz, in der Volhard sein großes Interesse an Alwalls Arbeit unterstreicht, kam Alwall in dem Manuskript auf den Urologenkongress zurück:

*„Der Urologenkongress in München 1949 versammelte Teilnehmer aus allen Teilen Deutschlands und den Nachbarländern. Den zugereisten Schweden war es eine fast überwältigende Manifestation der Kraft und des Lebenswillens der Wissenschaft in der zerbombten Stadt. Aus dem Schlachtfeld spross wieder das Grün. Volhard war Ehrengast des Kongresses und sein Auftreten entsprach auch dieser Würde […] Der Nestor war ein faszinierender Akteur mit sicherlich genau berechneter Distanz zu seinem bewundernden und dankbaren Publikum“* [19].

Nach Halle eingeladen hatte ihn Harald Dutz (1914–2010), einer der führenden Nephrologen der DDR und ein langjähriger Kooperationspartner Alwalls. Dutz hatte im Jahre 1958, also nur ein Jahr nach Alwall, in Rostock eine internistische Professur mit dem Schwerpunkt Nephrologie erhalten und damit ebenso wie Alwall zur universitären Etablierung des Fachs Nephrologie im jeweiligen Land beigetragen [20]. Diese persönlichen Bande über die Ostsee hinweg zwischen Lund und Rostock führten u. a. dazu, dass Alwall 1961 von der Universität Rostock die Ehrendoktorwürde verliehen wurde. Der Wissenschaftsaustausch mit schwedischen Forschern gewann nach dem Mauerbau im August 1961 und der damit verbundenen Isolation der DDR-Wissenschaft noch weiter an Bedeutung [21]. Noch im April 1961 war eine gesamtdeutsche Gesellschaft für Nephrologie in Wiesbaden gegründet worden, an deren Gründung auch Dutz beteiligt gewesen war. Das besondere Ziel der Gesellschaft sollte sein, Grundlagenforschung enger mit klinischer Anwendung zu verbinden [22].

Im Jahre 1965 erhielt Alwall die Ehrenmitgliedschaft der Gesellschaft für Innere Medizin der DDR. 1971 wurde der VIII. Kongress der Europäischen Dialyse- und Transplantationsgesellschaft (EDTA) in Ost-Berlin, „Hauptstadt der DDR“, gemeinsam von Alwall und Dutz organisiert [23]. An der Tagung nahmen mehr als 700 Wissenschaftlerinnen und Wissenschaftler aus 35 Ländern teil, wie offiziellen Presseorgane der DDR stolz betonten [24].

Geehrt wurde Alwall auch in Westdeutschland. Die Deutsche Gesellschaft für Nephrologie vergibt seit dem Jahr 1984 den Nils-Alwall-Preis „zur Förderung herausragender Wissenschaftlerinnen/Wissenschaftler auf dem Gebiet der Klinischen Nephrologie“. Über den engeren Forschungsdiskurs hinaus ist Alwall in Deutschland im öffentlichen Raum allerdings kaum präsent. Eine kleine Straße im schleswig-holsteinischen Ort Uetersen und eine nephrologische Praxis in Berlin sind nach ihm benannt.

Einen festen Platz im fachkulturellen Gedächtnis (nicht nur) der deutschen Nephrologie sicherten Alwall v. a. die Publikationen seiner später selbst einflussreichen akademischen Schüler und wissenschaftlichen Weggefährten. Der bereits erwähnte Carl M. Kjellstrand, zwischen 1957 und 1964 bei Alwall als Nephrologe ausgebildet und später Professor of Medicine and Surgery an der University of Minnesota, verfasste nicht nur Nachrufe auf Alwall [1], sondern auch mehrere Abhandlungen zur Geschichte der Dialyse, in deren Mittelpunkt wiederum sein schwedischer Lehrer stand [11, 25]. Horst Klinkmann (geb. 1935; [26]), der seine Facharztausbildung u. a. bei Harald Dutz in Rostock und Nils Alwall in Lund absolviert hatte und maßgeblich am flächendeckenden Aufbau der Dialyse in der DDR mitwirkte, stellte 2005 in einem Beitrag die „Bedeutung von Nils Alwall bei der Einführung der Hämodialyse und Ultrafiltration im europäischen Raum“ dar [27]. Eine ausführliche Biographie widmete ihm Håkan Westling (geb. 1928), ab 1967 Professor für Physiologie und später Rektor der Universität Lund [5]. Dass die aktive Erinnerung an Alwall sich inzwischen von der Zeitzeugenschaft gelöst hat, zeigen die Abhandlungen von Jan Kurkus, der als Professor an der Alwall-Klinik erst ab 1985 wirkte [3, 16].

In allen diesen Abhandlungen finden die Lunder Kollegen Alwalls allenfalls in den Verweisen zu den Originalpublikationen Erwähnung. Dass die Lunder Dialyseforschung aber keineswegs eine „One-Man-Show“ war und auch Forschungsimpulse aus Ostseeländern jenseits des Eisernen Vorhangs erhielt, kann an den beiden Emigranten Lembit Norviit und Adolfs Martins Steins demonstriert werden.

## Lembit Norviit – von Tartu nach Lund

In seinem Nekrolog für Lembit Norviit aus dem Jahr 1967 zeichnete der ehemalige estnische Botschafter in Schweden, Heinrich Laretei, das Bild eines bescheidenen und selbstlosen Arztes:

*„[…] schon lange verbindet man nicht mehr den Namen von Norviit mit der künstlichen Niere. Diese Ehre ging an die Schweden. Ich habe einmal Dr. Norviit darüber gefragt und er antwortete: ‚Die künstliche Niere ist im Einsatz und hat vielen Menschen das Leben gerettet. Wer sie erfunden hat, ist weder dem Patient oder dem Arzt überhaupt nicht wichtig‘. Diese Antwort war sehr charakteristisch für Dr. Norviit“* [28].

**Abb. 3:**
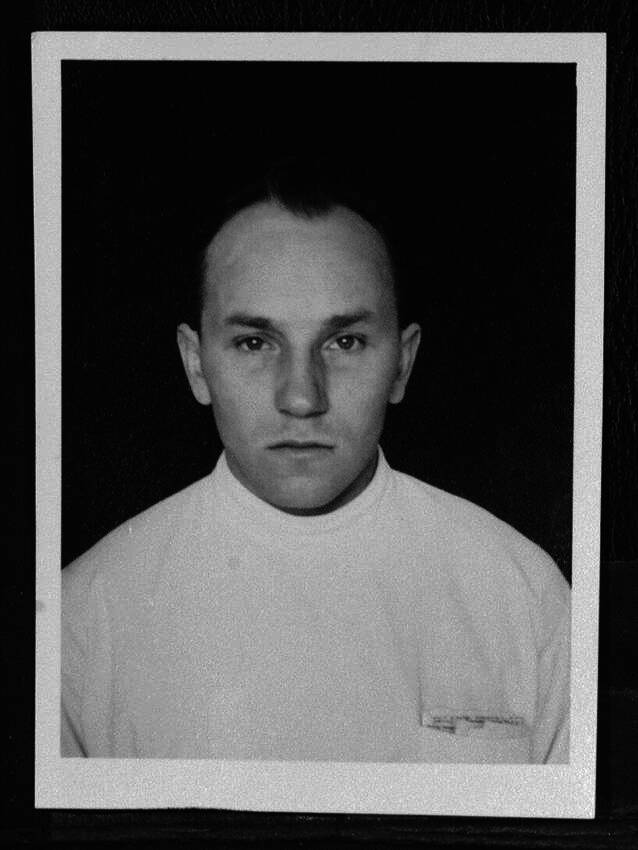
Lembit Norviit (1913–1967) im Mai 1941, RA, ERA.495.7.3633, Bl. 1. (Repro Tammiksaar mit freundl. Genehmigung Estn. Nationalarchiv)

Norviit (Abb. 3) wurde am 5. Juli 1913 in Helsinki geboren, wo sein Vater als Kanzleibeamter tätig war. Im Frühjahr 1918 siedelte die Familie nach Tallinn über. Dort legte Norviit 1932 sein Abitur ab. Nach einem Jahr im Heeresdienst begann Norviit im Jahr 1933 an der Universität Tartu sein Medizinstudium, das er 6 Jahre später mit cum laude beendete. Schon als Student forschte er 1937–1938 in der Klinik für Innere Medizin der Universität Tartu und erhielt den ersten Studentenpreis für seine Schrift „Askorbiinhappe eritamisest türeotoksikooside korral“ (Zur Ausscheidung von Ascorbinsäure bei Thyreotoxikose [29]^1^) im Dezember 1938 [30]. Weitere Auszeichnungen folgten. Von der Stadt Tallinn erhielt Norviit 1939 ein Stipendium [31].

Ab Oktober 1939 arbeitete Norviit als Volontärassistent in der I. Klinik für Innere Medizin in Tartu. Im März 1940 wurde er durch das Sozialministerium verpflichtet als Abteilungsarzt auf der Insel Ösel zu arbeiten. Erst ein halbes Jahr später konnte er seine Arbeit in der I. Klinik wieder aufnehmen, da er die Dysenterieepidemie auf der Insel bekämpfen musste [32]. Inzwischen war die Republik Estland von sowjetischen Truppen besetzt worden. Im Januar 1941 wurde Norviit zum Assistenten am Lehrstuhl für Innere Medizin an der Staatlichen Universität Tartu ernannt und übernahm dort die Leitung der Tuberkuloseabteilung [33].

Am 22. Juni 1941 begann offiziell der Krieg zwischen dem nationalsozialistischen Deutschland und der Sowjetunion. Zwei Monate später wurde Tartu von deutschen Truppen besetzt. Die Lehrtätigkeit an der Universität konnte erst im Januar 1942 aufgenommen werden. Norviit war wieder als Assistent an der I. Klinik tätig [34]. Anfang 1942 wurde er wieder, diesmal durch die von den deutschen Besatzern eingesetzte estnische Regierung, als Arzt nach Narva kommandiert, um dort die Dysenterie- und Fleckfieberepidemie zu bekämpfen. Danach wechselte er in ein Lager für Kriegsflüchtlinge in der Stadt Paldiski [35], bevor er ab Juli 1942 wieder seine Arbeit in Tartu aufnehmen konnte [36]. Am 15. April 1943 erklärte sich die Universität Tartu als im Krieg befindlich, sowohl mit der Sowjetunion als auch mit den westlichen Mächten. Als in der Aula der Universität die kriegswichtigen Forschungsthemen verkündet wurden, wurde Norviits Forschungsthema *Das Fleckfieber und die Störungen im Blutkreislauf* mit Prädikat „A“ ausgezeichnet [37]. Im Oktober 1943 wurde Norviit zum „älteren Inspektor“ zu Epidemien des Estnischen Gesundheitswesens vom Innendirektorium ernannt [38]. Offensichtlich bekleidete er diese Stelle bis zum 18. September 1944, als die estnische Selbstregierung ihre Arbeit in Tallinn beendete. Norviit flüchtete nach Ösel und von dort aus Anfang Oktober 1944 weiter über die Ostsee auf die schwedische Insel Gotland [39].

**Abb. 4:**
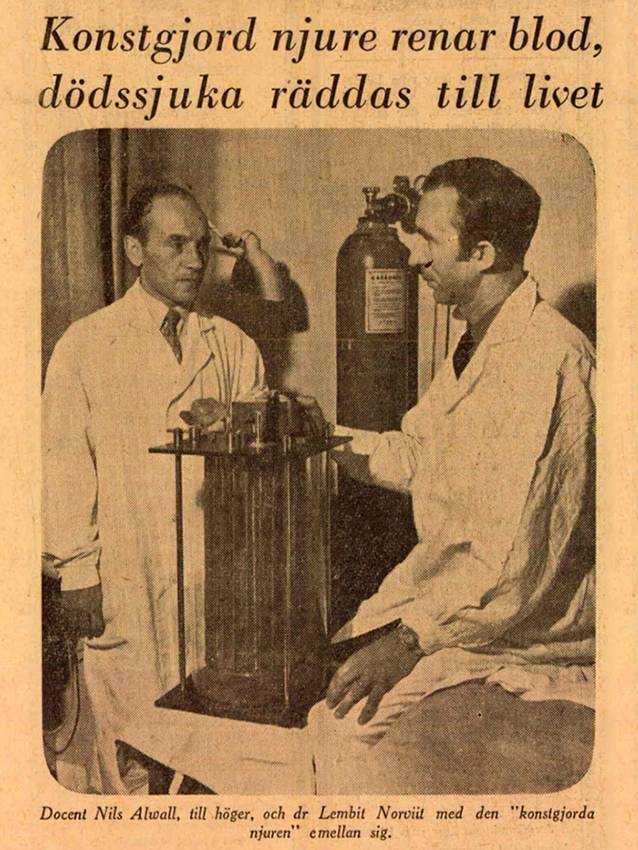
Dozent Nils Alwall und Dr. Lembit Norviit. (Aus: Dagens Nyheter 28.09.1947 mit freundl. Genehmigung)

Wie es dazu kam, dass er sich unmittelbar nach der Ankunft in Schweden mit Holzmodellen der künstlichen Niere befasste, ist nicht überliefert [40], aber schon wenige Monate nach der erzwungen Emigration begann er in Lund „als Archivar in der Inneren Klinik der Universität Lund“, wie er selbst schrieb [41]. Diese Angabe lässt sich mit archivalischen Quellen nicht belegen: Aus dem Lunder *Regionarkiv* geht lediglich hervor, dass Norviit als Assistent von Alwall zwischen Mitte 1949 bis Ende 1950 registriert worden ist. Allerdings zeigt eine Abbildung in „Dagens Nyheter“ bereits im September 1947 Arwall mit Norviit vor einer „künstlichen Niere“ (Abb. 4).

Alwalls erster Aufsatz über die Dialyse wurde 1947 publiziert [42]. Nachfolgende Artikel zu diesem Thema wurden mit der Beteiligung von Norviit veröffentlicht [43–48], einen publizierte Norviit auch separat [49]. Dank dieser Kooperation konnte Norviit im Jahr 1950 mit Alwall als Betreuer über ein Dialysethema [50] an der Universität Lund promovieren und damit seine bereits in Estland begonnene Forschungskarriere erfolgreich fortsetzen [51]. Seine Forschungsthemen lassen sich im Sinne eines Wissenstransfers allerdings nur schwer mit seinem Forschungsschwerpunkt in Lund übereinbringen. Warum Norviit nach der Promotion Lund verließ, um nach Nordschweden zu gehen, ist bisher nicht abschließend geklärt. Offenbar endete sein befristeter Arbeitsvertrag ohne erkennbare Aussicht auf Verlängerung [52].

Norviit wechselte in den Bereich der Arbeits- und Sozialmedizin, blieb aber trotz der praktischen Arbeit auch seinem Forschungsdrang treu. In den Jahren 1951–1952 war Norviit als Arzt im Sanatorium Solliden Nr. 114 in Östersund und danach in den Jahren 1952–1953 im Krankenhaus in Gällivare tätig. Zwischen 1953 und 1966 arbeitete er als Arzt und Forscher für das schwedische Bergbauunternehmen Boliden Gruv AB und befasste sich mit der Berufskrankheit Silikose. In der Zeitschrift *Smältdegeln* (Der Schmelztiegel) wurde ein langer Aufsatz über ihn publiziert, auch weitere schwedische Zeitungen schrieben über seine Silikosearbeit [53], die er in wissenschaftlichen Aufsätzen erläuterte [54–56] und auf Kongressen präsentierte, etwa in New York 1960 und in Madrid 1963 [57]. Norviit starb am 10. Februar 1967 im Alter von 54 Jahren.

Ganz vergessen ist Lembit Norviit zumindest in seiner alten Heimat nicht. Im biographischen Lexikon der estnischen Wissenschaft von 2013 heißt es u. a.:

„[Er] war 1944–50 Assistent an der Klinik für innere Krankheiten der Universität Lund, entwickelte die Technik der extrakorporalen Dialyse. Er entwickelte ein Gerät, das den Weg für die Entwicklung künstlicher Nieren in Schweden ebnete“ [58]^2^.

### Ādolfs Mārtiņš Šteins und seine berufliche Zwischenstation in Lund

Anders als für Norviit sind für Adolfs Martins Steins, geboren am 23. März 1911 in Vidriži/Lettland, einige Egodokumente überliefert, die seinen Werdegang erklären, vor allem auch warum die vielversprechende Forscherkarriere in Lund nur eine Zwischenstation blieb.

**Abb. 5:**
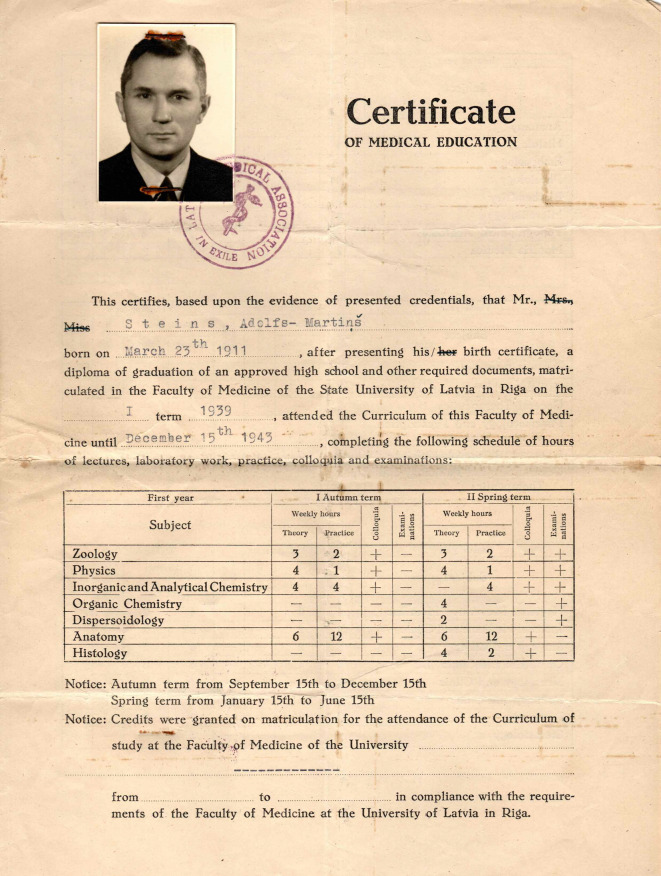
Abschlusszeugnis Adolfs Martins Steins 1944, ausgestellt von der Medizinische Fakultät der Universität Riga „– im Exil“ 1949. Privatbesitz. (Repro Juris Salaks, mit freundl. Genehmigung)

Steins begann sein Studium an der Medizinischen Fakultät der Lettischen Universität in Riga im Jahr 1939 und schloss es im März 1944 ab, 2 Monate früher als üblich aufgrund der Kriegsbedingungen (Abb. 5)^3^.

Davor hatte er bereits eine Ausbildung am Rigaer Lehrerinstitut absolviert und 8 Jahre lang als Lehrer an der Volksschule in der Gemeinde Livani gearbeitet. Bereits 1941 wurde die sowjetische Besatzung in Lettland durch die deutsche ersetzt. Die Situation an den Bildungseinrichtungen normalisierte sich, obwohl die direkten Kriegsdrohungen näher rückten. Die Memoiren von Steins über sein Studium während der deutschen Besatzung sind erhalten geblieben:

*„Ein deutscher Major (wahrscheinlich ein politischer), der am Hörsaal vorbeiging, hatte bemerkt, dass der Professor noch nicht gekommen war. Er machte ein paar Schritte in den Saal, um uns mit dem üblichen Hitlergruß zu begrüßen. Wir antworteten, wie wir es gewohnt waren, den Professor zu grüßen, indem wir mit dem Fuß den Boden berührten. Er verließ den Raum, weil er es ganz anders verstanden hatte. Unser Kursleiter und der Dekan der Fakultät mussten eingreifen, um den Major davon zu überzeugen, dass wir so auf Begrüßungen reagieren und dass wir nicht die deutschen Behörden und nicht die Armee beleidigen wollten“* [59].

Im Jahre 1944 erhielt Steins seine erste ärztliche Anstellung und wurde vom Leiter der lettischen Ärztekammer, E. Bush, als freier Arzt in die Region Grobiņa im Westen Lettlands geschickt. Von morgens bis abends, so berichtete er später, fuhr er auf einem Bauernkarren umher und besuchte seine Patienten. Doch der Aufenthalt im Kreis Grobina war nicht von langer Dauer. Fast alle Studienkollegen wurden zur Lettischen Legion eingezogen. Steins wurde entlassen, angeblich weil er nach der Wehrpflicht seine rechte Niere verloren hatte. Er bewarb sich trotzdem für die Legion und wurde aufgenommen. Die Angehörige dieser der deutschen Waffen-SS angeschlossenen Einheiten wurden in der sowjetischen Zeit als Kollaborateure verfolgt, gelten in jüngerer Zeit aber auch als lettische Freiheitskämpfer gegen den Bolschewismus [60]. Als Legionsarzt arbeitete Steins in Liepaja und versorgte die dortigen Militäreinheiten und Flüchtlinge bis zum 8. Mai, also bis zum Tag der deutschen Kapitulation [61]. Am gleichen Tag flüchtete er mit einem Boot von Lettland nach Schweden.

Unter den zahlreichen Flüchtlingen, die beschlossen, außerhalb Lettlands Zuflucht zu suchen, waren antisowjetische, nationalistische Politiker und Intellektuelle, die wussten, dass der Sieg Moskaus sie unmittelbar bedrohen würde [62]. Viele von denjenigen, die in Lettland blieben, wurden nach Sibirien deportiert, von wo nur ein Teil zurückkehrte. Diejenigen, die in den Westen emigrierten, verloren jedoch die Unterstützung ihrer Verwandten und Freunde. Es war ihnen nicht erlaubt, mit ihren Landsleuten in der Heimat zu kommunizieren. Sie waren für das Volk und die Gesellschaft Lettlands bis zum Dritten Erwachen („Trešā atmoda“) verloren, das durch Gorbatschows „Perestroika“ Mitte der 1980er-Jahre eingeleitet wurde und mit der Wiedererlangung der Unabhängigkeit Lettlands 1991 endete [63].

**Abb. 6:**
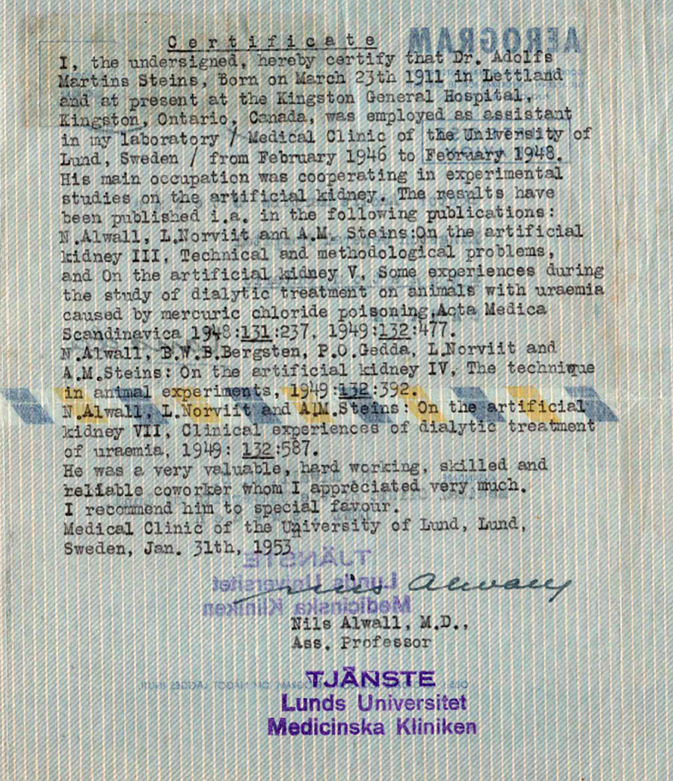
Schreiben Nils Alwall an Adolfs Martins Steins, Kingston, Ontario (Canada) vom 31.01.1953. Privatbesitz, Repro Juris Salaks, mit freundl. Genehmigung

Auch bei Steins konnte nicht geklärt werden, unter welchen Umständen er an die Universität Lund kam und die Zusammenarbeit mit dem damaligen Dozenten Alwall 1946 begann, zumal er, anders als Norviit, keinerlei Erfahrung in der medizinischen Forschung vorweisen konnte. Seine konkreten Beiträge für die Dialyseforschung bleiben weitgehend unklar. In einem Empfehlungsschreiben, das Alwall seinem ehemaligen Mitarbeiter 1953 ausstellte, beschrieb er dessen Tätigkeitsfeld: „His main occupation was cooperating in experimental studies on the artificial kidney“. Alwall verwies zudem auf Steins Mitautorschaft an 5 Beiträgen zu diesem Thema und hob, wie es für diesen Texttypus nicht unüblich ist, Steins Leistungsbereitschaft hervor: „He was a very valuable, hard working, skilled and reliable coworker whom I appreciated very much“ ([64]; Abb. 6).

Ab 1948 wirkte Steins dann jeweils für kurze Zeit als Arzt an Sanatorien und Krankenhäusern in Sundsvall, Vejbystrand und Söderhamn [65]. Nachvollziehbar ist auch Steins politisches Engagement für lettische Interessen. Als Vorsitzender der Lettischen Gesellschaft war er einer der Initiatoren der Idee einer lettischen Nationalstiftung in Schweden. Über das *Lettiska hjälpkommitten* blieb er auch mit seinen lettischen Studienkollegen in Kontakt [66].

Am 30. September 1951 heiratete er die ebenfalls aus Lettland stammende Krankenschwester Vilma Mandiane (1920–2000). Beide hatten ihre Heimat im Mai 1945 mit demselben Schiff verlassen. Im Herbst 1951 beschloss das junge Ehepaar nach Kanada auszuwandern. Diese erneute Emigration bedeutete, wieder eine neue Sprache lernen und für Steins die an der Universität von Lettland erworbenen medizinischen Kenntnisse wiederholen zu müssen, um als Arzt in Kanada arbeiten zu können.

In den Jahren 1953/1954 wirkte er als Forschungsstipendiat im traditionsreichen General Hospital an der Queen’s University in Kingston. Im Juli 1954 trat er in den Staatsdienst im Sudbury Gefängniskrankenhaus in Burwash, Ontario, ein [67]. Später zog er mit seiner Familie nach Bolton in der Nähe von Toronto um, wo er eine private Allgemeinarztpraxis eröffnete [68]. Er war Mitglied in vielen medizinischen Berufsverbänden, u. a. auch in der Vereinigung lettischer Ärzte und Zahnärzte im Ausland [69]. In Kanada setzte er auch seine Mitgliedschaft in der Studentenverbindung Fraternitas Lataviensis fort. Mehrere Jahre lang war er Vorstandsmitglied im Rat der National Association of Latvians of Canada. In der politischen Arbeit war Steins ein entschiedener Verfechter der lettischen Verfassung von 1922 und hat wiederholt die Notwendigkeit einer lettischen Exilregierung zum Ausdruck gebracht. Auf seine Initiative hin wurde 1973 der Freiheitsfonds gegründet [70], der die Hauptfinanzierungsquelle für die politische Arbeit der ausgewanderten Letten und für die Unterstützung der Wiederherstellung der Unabhängigkeit Lettlands war. 1986 beschloss der Rat der lettischen Nationalverbände in Kanada, den Vorstand des Weltverbands der Freien Letten aufzufordern, Steins für den lettischen Drei-Sterne-Orden vorzuschlagen [71].

Sein Heimweh zeigt sich in seinem Gedichtband *Voice of Blood* 1984, den er unter dem Pseudonym Kaspars Akmens veröffentlichte:

„Es pārbristu pāri pār jūru.

Lai dzimtenei sveicienus nestu,

Ja viļņi kā kalni būtu

Un jūra kā tuksnesis plašais.

Tad apkamptu dzimteni rokām

Kā māti skūpstītu mīļi.

[I wade over the sea.

To bring greetings to the motherland,

If the waves were like mountains

And the sea is as vast as the desert.

Then I would embrace the motherland in my arms

I would kiss like a mother with love]“ [72].

Steins starb am 9. August 1996 im Krankenhaus von Barrie in Kanada [73].

## Zusammenfassung für die Praxis

Im fachkulturellen Gedächtnis der deutschen und internationalen Nephrologie und Urologie ist Nils Alwall sehr präsent. Die Konstruktion einer der ersten in der Praxis erfolgreich eingesetzten Dialysemaschinen ist eng mit dem Wirken des schwedischen Mediziners verbunden. Zusammen mit Kollegen entwickelte er in den 1940er-Jahren einen Dialysator, der die Kombination von Dialyse und Ultrafiltration mit Membranen (Cellophan-Schläuchen) umsetzen konnte. Alwalls Koautoren in insgesamt 6 Beiträgen zu diesem Thema, die Mediziner Lembit Norviit aus Estland und Adolfs Martins Steins aus Lettland, sind auch in ihren Heimatländern weitgehend in Vergessenheit geraten. Als Emigranten verloren sie weitgehend die Bindung an ihr Heimatland, auch wenn etwa Steins in der politischen Bewegung des Exilletten engagiert war. Beide verließen – vermutlich auch aufgrund der befristeten Anstellungsverhältnisse – sehr schnell die Forschung wieder bzw. wandten sich im Falle Norviits anderen Themen zu und erhielten dort durchaus Anerkennung. Wichtige Voraussetzungen für den Eingang in das fachkulturelle Gedächtnis einer Disziplin, etwa die Ausbildung von akademischen Schülern oder dauerhafte soziale Netzwerke in der Scientific Community, wie sie für Alwall nachgezeichnet werden konnten, fehlten Norviit und Steins völlig.

In Hinblick auf den Wissenstransfer in Nordeuropa in der Zeit des Zweiten Weltkrieges und des Kalten Krieges sind Alwalls Kontakte nach Ostdeutschland durchaus charakteristisch für die besonderen Möglichkeiten, die sich auch der schwedischen Neutralität ergaben. Hingegen kann das Wirken von Lembit Norviit und Adolfs Martins Steins während ihres Forschungsaufenthalts bei Nils Alwall an der Universität Lund in dieser Hinsicht nur schwer beurteilt werden. Während Norviit Forschungserfahrungen in der Inneren Medizin besaß, ist bei Steins aufgrund seiner kriegsbedingten, ausschließlich praktischen medizinischen Erfahrungen nach Abschluss des Studiums keinerlei Wissenstransfer erkennbar. Im Gegenteil, beide haben sich ihre Kenntnisse in der Dialyseforschung, angeleitet von Alwall, erst in Lund angeeignet. Auch kehrten beide nicht bzw. erst nach dem Ende ihrer beruflichen Laufbahn in ihre Heimatländer zurück. Eine Koautorschaft eines *The Lancet*-Artikels, zumindest aus heutiger Sicht ein wichtiger Indikator für die vom „impact“ der jeweiligen Fachzeitschriften abhängige Anerkennung in den Wissenschaften, genügt, so verdeutlicht das Beispiel von Norviit und Steins, weder für eine dauerhafte Forscherkarriere, noch für eine Verankerung im fachkulturellen Gedächtnis einer medizinischen Fachdisziplin.

## Footnotes

1 Diese Forschungsarbeit wurde 1939 auf Deutsch publiziert.

2 Im Original: *„[…] oli 1944–50 Lundi Ük‑i sisehaiguste kliiniku asst, konstrueeris kehavälise dialüüsi aparaadi, mis pani aluse tehisneeru arengule Rootsis“*.

3 Certificate of Medical Education. Adolfs Martins Steins family archive held by his sons Namejs-Grant Steins (U.S.A.).
